# Viral MicroRNAs Encoded by Nucleocapsid Gene of SARS-CoV-2 Are Detected during Infection, and Targeting Metabolic Pathways in Host Cells

**DOI:** 10.3390/cells10071762

**Published:** 2021-07-12

**Authors:** Fei Meng, Gilman Kit-Hang Siu, Bobo Wing-Yee Mok, Jiahong Sun, Kitty S. C. Fung, Jimmy Yiu-Wing Lam, Nonthaphat Kent Wong, Lealem Gedefaw, Shumeng Luo, Thomas M. H. Lee, Shea Ping Yip, Chien-Ling Huang

**Affiliations:** 1 Department of Health Technology and Informatics, The Hong Kong Polytechnic University, Kowloon, Hong Kong, China; fei.meng@polyu.edu.hk (F.M.); gilman.siu@polyu.edu.hk (G.K.-H.S.); jiahong.sun@connect.polyu.hk (J.S.); kent.n.wong@connect.polyu.hk (N.K.W.); lealem.bimerew@connect.polyu.hk (L.G.); shumeng.luo@polyu.edu.hk (S.L.); 2 Department of Microbiology, The University of Hong Kong, Hong Kong, China; bobomok@hku.hk; 3 State Key Laboratory for Emerging Infectious Diseases, The University of Hong Kong, Hong Kong, China; 4 Department of Pathology, United Christian Hospital, Kwun Tong, Hong Kong, China; fungsck@ha.org.hk; 5 Department of Clinical Pathology, Pamela Youde Nethersole Eastern Hospital, Chai Wan, Hong Kong, China; lyw543a@ha.org.hk; 6 Department of Biomedical Engineering, The Hong Kong Polytechnic University, Kowloon, Hong Kong, China; ming-hung.lee@polyu.edu.hk

**Keywords:** MicroRNA, SARS-CoV-2 infection, host target genes, cellular metabolism, COVID-19

## Abstract

MicroRNAs (miRNAs) are critical regulators of gene expression that may be used to identify the pathological pathways influenced by disease and cellular interactions. Viral miRNAs (v-miRNAs) encoded by both DNA and RNA viruses induce immune dysregulation, virus production, and disease pathogenesis. Given the absence of effective treatment and the prevalence of highly infective SARS-CoV-2 strains, improved understanding of viral-associated miRNAs could provide novel mechanistic insights into the pathogenesis of COVID-19. In this study, SARS-CoV-2 v-miRNAs were identified by deep sequencing in infected Calu-3 and Vero E6 cell lines. Among the ~0.1% small RNA sequences mapped to the SARS-CoV-2 genome, the top ten SARS-CoV-2 v-miRNAs (including three encoded by the *N* gene; v-miRNA-N) were selected. After initial screening of conserved v-miRNA-N-28612, which was identified in both SARS-CoV and SARS-CoV-2, its expression was shown to be positively associated with viral load in COVID-19 patients. Further in silico analysis and synthetic-mimic transfection of validated SARS-CoV-2 v-miRNAs revealed novel functional targets and associations with mechanisms of cellular metabolism and biosynthesis. Our findings support the development of v-miRNA-based biomarkers and therapeutic strategies based on improved understanding of the pathophysiology of COVID-19.

## 1. Introduction

MicroRNAs (miRNAs) are small non-coding RNAs that primarily act in post-transcriptional processes and bind hundreds of sites in the transcriptome through targeting the 3′ untranslational regions (3′UTR), 5′UTR, and coding regions of specific mRNAs or embedding within a particular gene [1,2]. These RNA molecules have been implicated in a variety of pathological disorders including cancers, different organ dysfunctions, and infectious diseases [3,4,5,6,7]. As key regulators of gene expression, miRNAs may provide insights into the pathological processes involved in disease progression and associated complications.

Viral genomes of both DNA and RNA viruses encode miRNAs (v-miRNAs) that are expressed in infected host cells and participate in the life cycle and cellular pathophysiology after infection [8]. V-miRNAs function as innate immune agonists through binding and activating retinoic acid-inducible gene I (*RIG-I*), resulting in interferon expression and further disruption of the replication and translation processes in infected host cells [9]. For instance, v-miRNAs encoded by the nucleocapsid (*N*) gene of severe acute respiratory syndrome coronavirus (SARS-CoV) play critical roles in regulation of lung pathology and proinflammatory cytokines (MCP-1, IL-6, and CXCL10) [10].

SARS-CoV-2, an enveloped and single-stranded positive-sense RNA virus [11], possesses 82% nucleotide homology with the SARS-CoV genome [12]. The SARS-CoV-2 genes encode multiple structural and accessory proteins that facilitate viral entry and invasion of host cells [13], and are involved in inflammasome activation, cell death, and humoral immune response [14,15]. Recent clinical and computational studies on coronavirus disease 2019 (COVID-19) have shown the presence of both virus and host miRNAs with potentially significant roles in disease pathogenesis [16,17,18], including cases of thrombosis [19]. Here, we identified novel v-miRNAs in SARS-CoV-2-infected cell lines by small RNA deep sequencing and validated their expression in patient specimens using reverse transcription (RT) and droplet-digital PCR (ddPCR). Target gene and transcriptional data analyses [20] revealed a v-miRNA-based regulation system that could provide a novel mechanism underlying the hijacking of cellular metabolic and biosynthetic pathways by SARS-CoV-2. Given the current lack of effective therapies and the spread of highly infective mutant SARS-CoV-2 strains worldwide [21], targeting these v-miRNAs and their interacting pathways should facilitate the identification of effective biomarkers and therapeutic strategies to counteract the course of disease progression in patients with critical COVID-19.

## 2. Materials and Methods

### 2.1. Cell Culture and Infection of SARS-CoV-2

SARS-CoV-2 clones, HK-95 (PANGO lineage B.43; accession number MT835143) and HK-405 (PANGO lineage B.1.36.27; accession number MW856793), were isolated from the nasopharyngeal aspirate of sequence-confirmed COVID-19 patients in Hong Kong [22,23]. The viral isolates were grown through Vero E6 cells (CRL-1586, ATCC) [22,23] in Dulbecco’s modified Eagle’s medium (DMEM) (Thermo Fisher Scientific, Waltham, MA, USA) supplemented with 10% fetal bovine serum (FBS) (Thermo Fisher Scientific, Waltham, MA, USA), 100 units/mL penicillin and 100 µg/mL streptomycin (1% P/S) (Thermo Fisher Scientific, Waltham, MA, USA). All experiments involving live SARS-CoV-2 were done in a Biosafety Level 3 (BSL-3) facility with strict adherence to the standard operating procedures. Calu-3 human airway epithelia cell line (HTB-55, ATCC) was cultivated in DMEM (Thermo Fisher Scientific, Waltham, MA, USA) supplemented with 10% FBS, 1% L-glutamine, and 1% P/S in incubator at 37 °C with 5% CO_2_. Calu-3 cells were infected with SARS-CoV-2 of different strains at a multiplicity of infection (MOI) of 0.1 and incubated at 37 °C, 5% CO_2_ for 48 h. A mock sample was used as a control.

### 2.2. Small RNA Library Preparation and Sequencing

Total RNA was isolated from v-miRNA-transfected Calu-3 and Vero E6 cells by using RNeasy mini kit (#74104, QIAGEN). For SARS-CoV-2-infected cells, total RNA was isolated by precipitation method in the BSL-3 laboratory. In brief, 2.5 × 10^5^ infected cells were lysed with 200 μL of RNAzol, then mixed with 80 μL of water treated with diethyl pyrocarbonate (DEPC) and the mixture was vortexed for 15 s. After 15 min of incubation at room temperature, the preparation was centrifuged at 12,000× *g* (at 4 °C) for 20 min. RNA was then precipitated from the aqueous layer with an equal volume of isopropanol at −20 °C and recovered by centrifugation (12,000× *g* at 4 °C for 30 min), washed once with 75% ethanol, air-dried, and dissolved in DEPC-treated water.

The concentration and quality of RNA were checked using Nanodrop spectrophotometer (Thermo Fisher Scientific, Waltham, MA, USA) and Qubit 2.0 Fluorometer (Thermo Fisher Scientific, Waltham, MA, USA), respectively. The integrity of the total RNA was analyzed by Agilent 2100 Bioanalyzer system and processed further when the RNA integrity number (RIN) was >7.0. Small RNA libraries were prepared from 100 ng of total RNA input using NEB Next^®^ Multiplex small RNA library preparation kits (#E7330S, NEB) following the manufacturer’s instructions. The small RNA libraries were prepared in both biological and technical triplicates. The RNAs were ligated with 3′ and 5′ RNA adapters, then RT, PCR enrichment with barcoded RT primers, and size selection were performed following manufacturer’s instructions. PCR amplification was done using the LongAmp Taq 2X master mix (NEB, USA), Index (X) primer and SR primer for Illumina, based on the following amplification conditions: 94 °C for 30 s, followed by 15 cycles of denaturation at 94 °C for 15 s, annealing at 62 °C for 30 s, extension at 70 °C for 15 s, and one cycle of final extension at 70 °C for 5 min. The PCR-amplified cDNA products were purified using Monarch PCR & DNA Cleanup Kit (#T1030, NEB). A 6% polyacrylamide gel was used for the library quality control (QC) and fragment size selection (approximately 140 bp). The filtered products were used for the construction of the final library. For size confirmation, 1 µL of size-selected purified library was loaded onto the Agilent 2100 Bioanalyzer system using a high-sensitivity DNA chip according to the manufacturer’s instructions. The library concentration was checked by KAPA library quantification kit (KK4824, Roche, Cape Town, WC South Africa). The next-generation sequencing (NGS) was conducted in Illumina Nova-seq platform.

### 2.3. Bioinformatics Analysis

FastQC was used to check the quality of sequencing reads and derive the original reads number [24]. Fastp was used to trim reads with adaptors (AAGATCGGAAGAGCACACGTCT and GATCGTCGGACTGTAGAAC), remove low-quality reads, and select the reads with length less than 30 bp and more than 15 bp [25]. Then, cleaned reads were mapped to SARS-CoV-2 reference genome (Wuhan-Hu-1 NC_045512.2) and exported to SAM files using BWA MEM [26]. GATK was used to sort and index the SAM files. Virus-genome-mapped reads were then aligned to human reference genome (hg38) to make sure all reads were uniquely mapped to the virus genome [27]. For the selection of v-miRNA, SAMtools was used to calculate the base read count, by which regions with higher read coverage than uniformly distributed background were preliminarily selected. Then IGV was used to visually inspect the top ten v-miRNAs with the highest read coverage, the consensus v-miRNA sequence was determined by the summit of the peak with length between 18 and 22 bp [28]. BEDTools was used to calculate the exact read count in each v-miRNA region and determine the percentage of v-miRNA reads among all mapped reads [29]. The secondary structure of v-miRNAs was predicted and visualized by RNAfold [30] and forna [31]. Prediction of potential target genes in host cells were performed by combining miRDB [32] and DIANA tools [33]—Strategy 1. Only the targets with scores over 70 were considered and the overlap targets predicted by both methods were treated as the reliable set [17]. For Strategy 2, seed regions (bases 2–7 from 5′ to 3′) were extracted from v-miRNAs and then searched in 3′UTR database of human genes for their reverse complementary sequence. The genes with matched sequence were selected as the potential targets of v-miRNAs. Overrepresentation analysis (ORA) of Gene Ontology (GO) was performed by using Panther and Panther GO-Slim BP annotation sets [34]. The putative targets of v-miRNAs were combined as the query list and the human whole gene list was set to be the reference list. Fisher exact tests with Bonferroni correction for multiple testing (cutoff 0.05) were applied in the analysis. Calu-3 and Golden hamster RNA-seq data were downloaded from GEO database, followed by DEseq2 [35] to analyze the differentially expressed genes (DEGs) between virus-infected group and mock-infected group. DEGs with |log2FoldChange| > 1 and FDR < 0.05 were selected to be de-regulated genes. For Vero E6, a table of DEGs was downloaded from relevant Supplementary Materials [20], and the same criteria were used to select de-regulated genes.

### 2.4. Reverse Transcription (RT) and Droplet-Digital PCR (ddPCR)

First-strand cDNA was produced using RevertAid first-strand cDNA synthesis kit (#K1622, Thermo Fisher Scientific, Waltham, MA, USA) with v-miRNA-N-specific RT primer (100 µM) (5′-GTCGTATCCAGTGCAGGGTCCGAGGTATTCGCACTGGATACGACCCAGCT-3′), which forms a stem-loop structure to achieve the reaction specificity. RNA sample (100 ng) was mixed with 1 µL of v-miRNA-N-specific RT primer and then heated at 65 °C for 5 min, followed by incubation on ice for 1 min. A master mix containing 5X first-strand buffer, dNTP Mix (10 mM), RiboLock RNase inhibitor (20 U/µL), and RevertAid M-MuLV RT (200 U/µL) was added to give a 20-µL reaction volume. The reaction mix was incubated at 16 °C for 30 min, 42 °C for 60 min, and 70 °C for 5 min. For ddPCR, a 20-µL reaction mixture was prepared, comprising 1 µL cDNA library sample, 10 µL 2X ddPCR EvaGreen Supermix (Bio-Rad, Hercules, CA, USA), 250 nM each of forward and reverse primers (Forward: 5′-GCCCGCTAGGAACTGGGCCAGAAG-3′; Reverse: 5′-GTGCAGGGTCCGAGGTAT-3′), and nuclease-free water. The reaction mix was loaded into the sample wells of the DG8 cartridge (Bio-Rad), and aqueous droplets in oil were generated using QX200 droplet generator (Bio-Rad). Droplet mixtures each of 40 µL were gently transferred into a 96-well plate. The sealed 96-well plate was subjected to following thermal cycling conditions in a thermocycler: 95 °C for 10 min, 40 cycles of 94 °C for 30 secs and 60 °C for 60 secs, followed by 98 °C for 10 min. Then the plate was analyzed using QX200 droplet reader (Bio-Rad, Hercules, CA, USA).

### 2.5. Transfection of Small RNA Mimics

Calu-3 cells and primary human peripheral blood mononuclear cells (PBMCs) were plated on 6-well plates. The cells were transfected with 50 nM v-miRNA-N mimics using Lipofectamine 2000 reagent (LF2000) (Thermo Fisher Scientific, USA) following the standard protocols. Briefly, cells were seeded into new 6-well plates after washing with Opti-MEM. LF2000 and v-miRNA-N mimics were diluted and mixed in Opti-MEM medium, and incubated at room temperature for 20–30 min to form transfection complex. The transfection complex was transferred into each well, and gently mixed with the cells. The plates were incubated at 37 °C and 5% CO_2_ for 48 h before collection of cells. After 48 h of incubation post-transfection, the cells were harvested and total RNA was isolated using RNeasy mini kit (QIAGEN). The quantity of miRNA isolation was detected by Qubit microRNA Assay Kit (Invitrogen, Thermo Fisher Scientific). The expression levels of transfected v-miRNA-N mimics were validated by NGS small RNA-seq and quantitative PCR after RT.

### 2.6. Reverse Transcription-Quantitative Polymerase Chain Reaction (RT-qPCR)

cDNA samples were prepared by RevertAid first-strand cDNA synthesis kit (#K1622, Thermo Fisher Scientific) as mentioned in Section 2.4, but oligo (dT)_18_ primer was used as the RT primer. qPCR was performed with the QuantiNova SYBR Green PCR Kit (Qiagen) following the manufacturer’s instructions. The reactions were conducted and detected using ViiA™ 7 Real-Time PCR system (Applied Biosystems) with the following thermal cycling conditions: 95 °C for 2 min, 40 cycles of 95 °C for 30 secs, and 60 °C for 60 secs, followed by the default melting curve analysis stage. The relative gene expression change was calculated by 2^−△△Ct^ method after normalization to the GAPDH expression (Forward primer: 5′-AGGTCGGAGTCAACGGATTTG-3′; Reverse primer: 5′-TGAAGG GGTCATTGATGGCAACA-3′) for protein-expressing transcripts; miRNA expression was normalized to the U6 expression (Forward primer: 5′-GCTTCGGCAGCACATATACTAAAAT-3′; Reverse primer: 5′-CGCTTCACGAATTTGCGTGTCAT-3′). The primer sequences for detecting the target genes are listed in Table S1.

### 2.7. Protein Extraction and Western Blotting

Total protein was extracted from the cells using RIPA lysis buffer (50 mM Tris-HCl, pH 7.4, 150 mM NaCl, 1% NP-40) containing protease and phosphatase inhibitor. Cells were lysed for 30 min on ice with occasional vortexing and then centrifuged at 15,000× *g* at 4 °C for 30 min. The extracted protein was quantified with the Pierce™ BCA Protein Assay Kit (Thermo Fisher Scientific) according to the manufacturer’s instructions. In total, 50 μg protein was resolved by 10% Bis-Tris polyacrylamide gels and transferred to polyvinylidene fluoride membrane. The membrane was blocked for 1 h in PBS containing 5% non-fat dry milk and 0.05% Tween-20, and probed with appropriate primary antibodies at 4 °C overnight. After washing with PBS containing 0.1% Tween-20, the membrane was incubated with secondary antibodies at room temperature for 1 h. Signals were visualized with enhanced chemiluminescence substrates. Antibody information is listed in Table S2.

### 2.8. Patient Samples and Ethics Approval

Samples of total nucleic acid isolated from COVID-19 patients’ nasopharyngeal aspirate were collected and stored with the approval of the Research Ethics Committees of the Hospital Authorities (Hong Kong) and The Hong Kong Polytechnic University, including the use of consent forms (HSEARS20210123003). All procedures in this research were carried out in compliance with the ethical principles of the institutional research and safety committee.

### 2.9. Statistical Analyses

Statistical analysis was performed using GraphPad Prism version 9.0 (GraphPad Software, San Diego, California USA). All results were reported as mean ± SD from at least three biological replicates. To evaluate differences between multiple groups, unpaired *t*-test or the non-parametric Mann-Whitney test were performed. *p* < 0.05 was considered as statistically significant.

## 3. Results and Discussion

### 3.1. Expression of Viral MicroRNAs Encoded by SARS-CoV-2 N Gene

**Figure 1:**
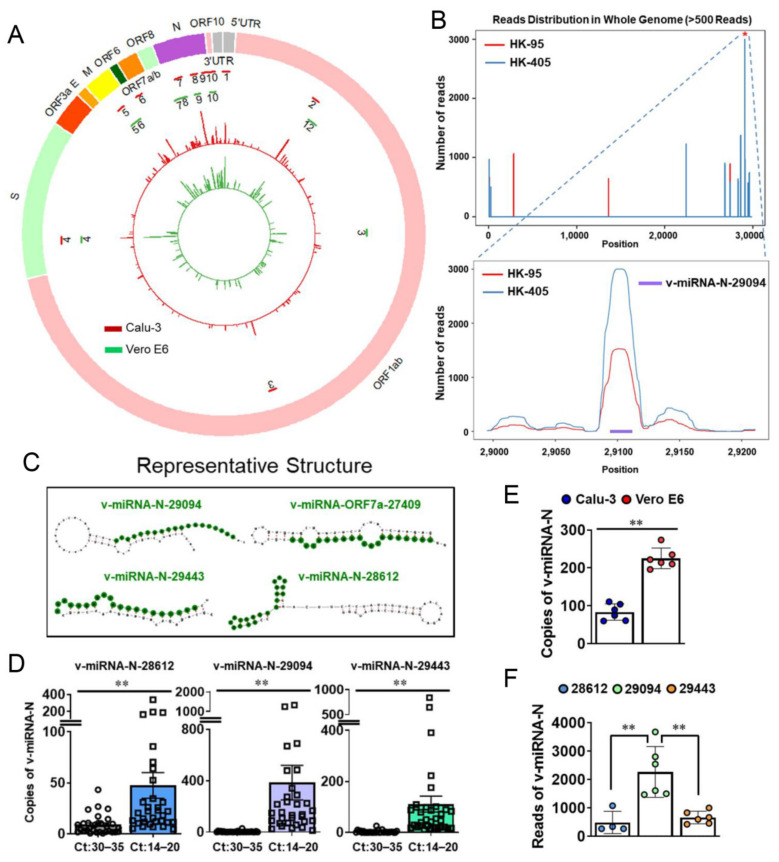
Detection of SARS-CoV-2-encoded v-miRNAs. (**A**) Genetic map and read distribution of SARS-CoV-2-encoded v-miRNAs in Calu-3 and Vero E6 cells. The inner green layer represents read distribution from Vero E6 cells and the middle red layer represents the read distribution of v-miRNAs from Calu-3 cells. The locations of the top ten v-miRNAs (in terms of read counts) are indicated as two circular layers of 10 short bars with the outer layer (red) for Calu-3 cells and inner layer (green) for Vero E6 cells. (**B**) Read distribution across the entire whole viral genome (>500 reads). The Y-axis shows the number of reads, and the X-axis shows the position of v-miRNAs. V-miRNA-N-29094 showed the highest peaks both in HK-95 and HK-405 samples isolated from Calu-3. (**C**) Representative predicted secondary structures of pre-miRNAs (gray). The encoded green color indicates the region for NGS-identified mature v-miRNAs. (**D**) Copy number of v-miRNA-N detected in COVID-19 patients. Significantly increased copy numbers of v-miRNA-28612, v-miRNA-229094, v-miRNA-29443 were detected in clinical samples from COVID-19 patients with high viral load (low Ct: 14–20) compared to those with low viral load (high Ct:30–35). The mean ± SD is shown for RT-ddPCR results. *n* = 30–33; ** *p* < 0.01. (**E**) Expression levels of v-miRNA-N-28612 are quantified by RT-ddPCR in Calu-3 and Vero E6 cells. The mean ± SD is shown for RT-ddPCR results. ** *p* < 0.01. (**F**) NGS reads of the identified three v-miRNA-N are determined in Calu-3 cells. The mean ± SD is shown for results obtained from small RNA-seq. ** *p* < 0.01. Ct, threshold cycle; E, envelope; HK, Hong Kong; M, membrane; N, nucleocapsid; ORF, open reading frame; S, spike; UTR, untranslated region; v-miRNA-N, viral microRNA nucleocapsid.

SARS-CoV-2 viral infection in both Calu-3 and Vero E6 cell lines were induced at 0.1 and 0.01 MOI (i.e., 10^5^ virions to Calu-3 and 10^4^ virions to Vero E6, for a total of 1 million cells each), respectively, for 48 h. Small RNAs obtained from infected cells were deep-sequenced after size selection. Approximately 0.1% of the small RNA sequences were mapped to the SARS-CoV-2 genome (Figure 1A,B), including the consensus region identified from the *N* gene of SARS-CoV (small viral RNA-N, svRNA-N; [10]) among the top ten v-miRNAs (in terms of read counts) encoded by SARS-CoV-2 (Figure 1A, Table S3). No significant reads were detected in mock-infected cells after validation with quality filtration of each sequence and background. Moreover, no similar sequences in the human genome could be mapped with the ten selected v-miRNAs.

We additionally analyzed the structural features of these ‘sequence-identified’ v-miRNAs and their positions in the respective regions of putative pre-miRNAs. The top three SARS-CoV-2 v-miRNAs encoded by the *N* gene (v-miRNA-N-28612, v-miRNA-N-29094, and v-miRNA-N-29443) were located mainly in the stem-like regions, as predicted with MatureBayes, RNAFold and forna [17,36] (Figure 1C). These ten v-miRNAs were identified in the *N* gene along with 5′ end of the viral genome (*5′UTR*), *Orf1ab*, *Orf2* (Spike; *S*), *Orf5* (Membrane; *M*), *Orf7a*, and *Orf10* of SARS-CoV-2, which mainly include the coding regions for structural and accessory proteins [13]. The most significant SARS-CoV-2 v-miRNAs were derived from the *N* gene (about 5.9% of total coverage) encoding the highly immunogenic nucleocapsid protein that elicits the main humoral immune response in COVID-19 patients [37,38]. Accordingly, we propose that potential biomarker development with addition of v-miRNA panels to targeted SARS-CoV-2 gene sequencing should improve COVID-19 diagnosis and prediction of the disease course [39].

### 3.2. V-miRNAs Derived from the N Gene Are Differentially Expressed in COVID-19 Patients

To study the expression of v-miRNA-N-28612 (the identified SARS-CoV svRNA-N; [10]) and determine its association with viral load in patients, RT-ddPCR was performed. Notably, samples isolated from nasopharyngeal aspirate were tested positive for svRNA-N (v-miRNA-N-28612, v-miRNA-N-29094, and v-miRNA-N-29443) (Figure 1D). Moreover, copy number analyses revealed a significant association with viral load/genomic RNA of SARS-CoV-2 in patient specimens (Figure 1D). Expression levels of different v-miRNAs may be host-cell specific, as reflected from the results obtained with Calu-3 and Vero E6 cells (Figure 1E, Table S3). Obtained from our NGS-captured experiment, and followed by sequencing and ddPCR validation, these data were significant and worthy of future investigations. Earlier evidence suggested that biogenesis of these v-miRNAs depended on the capacity of viral replication [10]. In addition, SARS-CoV-encoded svRNA-N has been shown to be associated with lung pathology in an in vivo experimental setting [10].

Copy numbers of v-miRNA-N reflected their quantity from the same sample sources and with a similar pattern detected in the NGS system and the highest reads for v-miRNA-N-29094 (Figure 1B,F). One limitation of the quantification method used is that we could not distinguish whether a small proportion of v-miRNA-N signals is coming from secreted vesicles instead of cellular components of the collected patient samples. This hypothesis will be further investigated using different specimen sources, such as blood plasma [10], which may additionally be associated with disease pathogenesis but not the controversial findings with viral load [40,41]. Moreover, we also proposed that there might be a tissue/cell-specific regulation of the v-miRNAs as well as their interactions with host genes. This hypothesis was based on the existence of tissue/cell-specific miRNAs (endogenous) investigated and therefore the v-miRNAs interacting with host target genes could be competed by these endogenous cellular miRNAs containing the similar binding regions. On the other hand, the expression level of SARS-CoV-2 RNA genome can also be regulated by cellular miRNAs. We expect that the same v-miRNAs can even undergo different changes in epithelial cells of other organs if there are differentially expressed miRNAs among them.

### 3.3. SARS-CoV-2 V-miRNAs Target Host Genes and Pathways Related to Cellular Metabolic and Biosynthetic Processes

**Figure 2:**
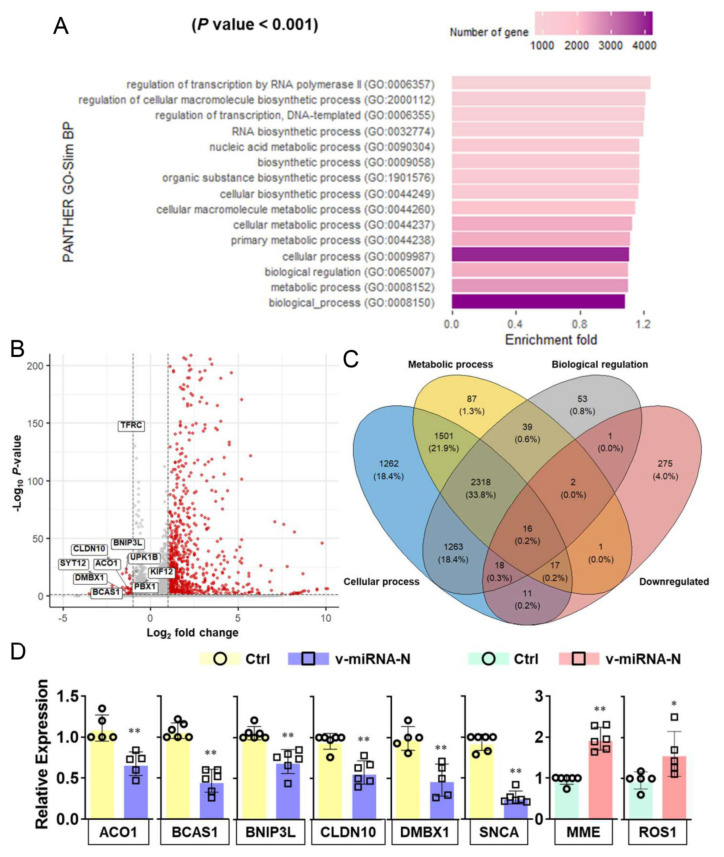
SARS-CoV-2-encoded v-miRNAs target host genes that are involved in metabolic pathways. (**A**) GO analysis indicates the main enrichment terms targeted by SARS-CoV-2-encoded v-miRNAs (predicted by using the seed-region method). (**B**) The volcano plot shows DEGs targeted by v-miRNAs. The red dots show significantly upregulated and downregulated genes (|log2FoldChange| > 1 and FDR < 0.05) while the grey dots show the remaining genes not satisfying these criteria. (**C**) A Venn diagram shows the numbers of genes overlapping between and among down-regulated genes and genes enriched in different GO pathways. Down-regulated genes are identified by edgeR with log_2_FoldChange less than −1 and FDR less than 0.05. Genes enriched in metabolic process, cellular process and biological regulation are predicted by the seed-region method. (**D**) RT-qPCR results demonstrate the relative expression of overlapping host target genes in Calu-3 cells transfected with negative control (Ctrl) or v-miRNA-N synthetic mimics. The five histograms on the left show downregulated genes while the two histograms on the right show upregulated genes. The mean ± SD is shown for RT-qPCR results. *n* = 5–6; * *p* < 0.05; ** *p* < 0.01. *ACO1*, aconitase 1; *BCAS1*, brain enriched myelin associated protein 1; *BNIP3L*, BCL2 interacting protein 3 like; *CLDN10*, claudin 10; Ctrl, control; *DMBX1*, diencephalon/mesencephalon homeobox 1; GO, gene ontology; *KIF12*, kinesin family member 12; *MME*, membrane metalloendopeptidase; *PBX1*, pre-B-cell leukemia transcription factor 1; *ROS 1*, ROS proto-oncogene 1, receptor tyrosine kinase; *SNCA*, synuclein alpha; *SYT12*, synaptotagmin12; *TFRC*, transferrin receptor protein 1; *UPK1B*, uroplakin 1B; v-miRNAs, viral microRNAs.

In silico analyses of SARS-CoV-2 v-miRNAs were further conducted to investigate the mechanisms underlying pathogenesis during COVID-19 progression. Over-representation of gene ontology revealed enriched terms of host target genes of all v-miRNAs based on regulation of biological processes. The host target genes for SARS-CoV-2 v-miRNAs were selected by mapping the reverse complement of their seed regions to 3′UTR database of human reference genes, followed by Panther GO-Slim biological process for functional enrichment (Figure S1A). A total of 15 human gene clusters were identified as potential targets of SARS-CoV-2 v-miRNAs. GO analysis further revealed that target genes were significantly enriched in the functions of cellular metabolic (GO:0044237, GO:0009987) and cellular biosynthetic (GO:0044249, GO:0009058) processes (Figure 2A). Interestingly, different significant pathways were identified with the two strategies [17] (Figure 2A and Figure S1B) while the seed region mapping approach revealed similar functional targets in cellular metabolism, as recently reported with metabolomics analysis in SARS-CoV-2 infected cells [20].

With further interpretation combined with RNA-seq data (#GSE161881 [20] and # GSE156005 [22]), we summarized that more host genes were up-regulated rather than down-regulated upon infection (Figure 2B). Since we hypothesized that v-miRNAs might directly interfere target gene expression (at a post-transcriptional level), we attempted to find the overlaps between the down-regulated host genes and predicted host targets that are enriched in metabolic processes. Overlapped genes include *TFRC*, *BNIP3L*, *ACO1*, *UPK1B*, *KIF12*, *CLDN10*, *DMBX1*, *SYT12*, *BCAS1*, *PBX1*, *MME*, *MMP11*, *ROS1*, *SNCA*, *BCAT1* (Figure 2B,C, Table S4). RT-qPCR analysis further demonstrated the down-regulated expression of host target transcripts *ACO1*, *BCAS1*, *BNIP3L*, *CLDN10*, *DMBX1*, and *SNCA* after the overexpression of v-miRNA-N (v-miRNA-N-28612) using synthetic mimics (Figure 2D).

ACO1 plays a role in iron metabolism by regulating the translation of iron metabolizing proteins to control cellular iron levels [42]. The *ACO1* gene is overexpressed in peripheral blood samples of COVID-19 patients and this may indicate a different response to immune activation [43]. *BCAS1*, a gene located in chromosome 20q13.2, was reported with a role in myelinating oligodendrocytes related to multiple sclerosis lesions [44]. *CLDN10* is a gene that encodes the claudin 10 protein and its mutations are linked to epithelial dysfunction with impaired ion transport [45]. In addition, *ACO1* and *CLDN10* were considered to be involved in regulating carbon metabolism and immune cell transmigration, respectively [46]. The direct binding effects of v-miRNA-N are under investigation using miR-CLIP-based approach [47].

### 3.4. Increased IL-1β, Caspase 1, and NLRP3 Expressions Were Detected after Transfection of v-miRNA-N Synthetic Mimics

**Figure 3:**
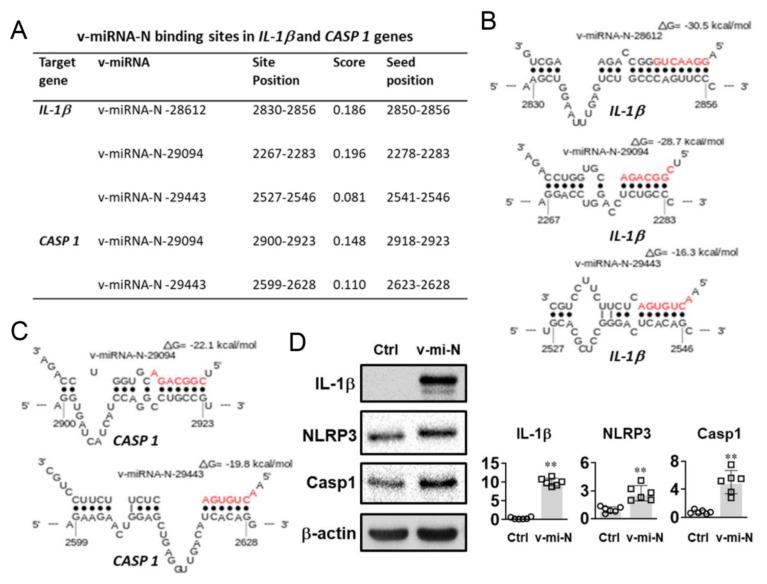
Putative interactions of SARS-CoV-2 v-miRNA-N were found in host genes related to inflammasome activation. (**A**) A summary of the binding sites of v-miRNA-N-28612, v-miRNA-N-29094, and v-miRNA-N-29443 in the 3′UTR sites of *IL-1β* and *CASP 1* gene. (**B**) and (**C**) show the binding sites for v-miRNA-N-29094 and v-miRNA-N-29443 in the 3′UTR regions of *IL-1β* and *CASP 1* gene, which are analyzed using Sfold STarMir (https://sfold.wadsworth.org/cgi-bin/starmirtest2.pl) (accessed on 25 May 2021). The seed sequences of v-miRNA-N species are shown in red. (**D**) Western blot results show the expression of IL-1β, NLRP3, and caspase 1 (Casp1) proteins in PBMCs transfected with negative control (Ctrl; leftside) or v-miRNA-N (v-mi-N; rightside) synthetic mimics. *n* = 6 (obtained from biological replicates); ** *p* < 0.01. *CASP 1*, caspase 1; *IL-1β*, interleukin one beta; NLRP3, nucleotide oligomerization domain-like receptors family pyrin domain containing 3; v-miRNA-N, viral microRNA nucleocapsid.

In addition to pathway analysis, putative binding sites of SARS-CoV-2 v-miRNA-N were found in host genes that are related to inflammasome activation, specifically IL-1β and caspase 1 (Figure 3A–C). Forced expression of v-miRNA-N (v-miRNA-N-28612) through liposome transfection in human PBMCs enhanced IL-1β, NLRP3, and caspase 1 protein expressions (Figure 3D). The increased expression of key inflammasome markers demonstrates the active role of SARS-CoV-2-derived v-miRNA-N in modulating innate immune processes [48]. The canonical process of inflammasome activation is dependent on caspase 1. Caspase 1 could convert proinflammatory cytokines into their functional counterparts, including mainly pro-IL-1β and pro-IL-18. Active caspase 1 further triggers pyroptosis, a form of cell death induced by inflammation, which can cause pathogenic tissue damage and cytokine storm if out of control [49]. The NLRP3 is the most well-known sensor molecule for virus and other microorganisms [49,50,51]. NLRP3 produces host cell damage and induces downstream immune responses to certain infections and environmental stimuli [52,53,54]. V-miRNAs have the potential to regulate these sensor molecules through a variety of methods. According to Haneklaus et al. [55], EBV miR-BART15 interferes the NLRP3 inflammasome and IL-1β secretion by targeting the miR-223 binding site in the NLRP3 3′UTR. Pyroptosis is another mechanism induced by SARS-CoV-2 and linked to inflammasome activation.

IL-1β is the most prevalent pro-inflammatory cytokine found in severe COVID-19 patients and is linked to cytokine storms [56]. The induction of IL-1β without other stimuli provides an evidence for the role of v-miRNA-N in activation of the inflammasome pathway. Previous findings indicated that SARS-CoV-2-encoded miRNAs may target NFκB, JAK/STAT, and TGF-β signaling pathways, which could drive expression of pro-inflammatory cytokines indirectly [48]. Similarly, svRNA-N encoded by SARS-CoV has been shown promoting inflammation in vivo through inducing the expression of pro-inflammatory responses and thus triggering lung pathogenesis [10]. These induction effects of v-miRNAs on inflammation pathways could be the downstream responses, which are derived from the competition with endogenous cellular miRNAs containing the similar binding regions. The detailed mechanistic insights are important because the alterations in v-miRNA levels have been implicated in the prediction of viral pathology, which will further contribute to early diagnosis and therapy.

Metabolic syndromes are identified as predictors in COVID-19-associated morbidity and mortality [57], which may be due to organ failures [58]. Molecular mechanism linking SARS-CoV-2 and metabolic alterations has been reviewed recently [59]. SARS-CoV-2 infection appears to be dependent on host micronutrients, particularly folates and purine biosynthesis precursors. SARS-CoV-2 infection alters host cellular metabolism of, e.g., intracellular glucose, amino acids, and folate during the early stage to fulfill the huge demand for ribonucleotide synthesis [20]. In addition, the enhanced purine synthesis allows more mRNA and virion to be synthesized and facilitates viral replication [20]. SARS-CoV-2 infection, on the other hand, causes anaerobic glycolysis by upregulating important glycolysis genes such as hexokinase 2 and pyruvate kinase isozyme, and causing dysregulation of the citric acid cycle [60]. The host target genes identified in our analysis were also found to be involved in iron metabolism (Figure 2B,D). Iron is an essential element in cellular oxygen transport and other metabolic activities. Gene dysregulation and impaired iron metabolism may be one of the factors associated with iron overload and high ferrittinemia observed in COVID-19 patients [61]. The presence of free and excess iron results in the generation of free radicals and damage to different tissues and organs [62]. On the other hand, a defect in the uptake and utilization of iron may lead to hypoferremia causing increased hospitalization and high oxygen demand [63] that leads to respiratory distress syndrome, the main clinical feature seen in patients with severe COVID-19 [64]. Hence, therapeutics targeting v-miRNA-N through anti-miRNA inhibitors coupled with personalized controls over metabolic and micronutrient status, and inflammasome activation, may provide significant contribution in limiting the COVID-19-associated pathogenesis.

## 4. Conclusions

We show here the first example of identifying SARS-CoV-2 v-miRNAs by an NGS sequencing approach and the subsequent experimental validation of some of their putative target genes in the host cells. The conserved v-miRNA-N-28612 was detected in SARS-CoV-2-infected cells and differentially expressed in COVID-19 patients with different viral loads. Combined target gene prediction and RNA-seq analyses led to the potentially novel mechanisms of v-miRNA-based regulation targeting cellular metabolic and biosynthetic pathways. Our data highlight the crosstalk between SARS-CoV-2 v-miRNAs and their host target genes, and contribute to mechanistic insights into the pathophysiology of COVID-19.

## Data Availability

All data generated and analyzed in this study are included in this article and its Supplementary Materials. The datasets of small RNA-seq presented in this study can be found in online repositories. The small RNA-seq raw data can be found in the NCBI’s Sequence Read Archive (SRA) with accession number PRJNA733311.

## Supplementary Materials

The following are available online at https://www.mdpi.com/article/10.3390/cells10071762/s1, Figure S1: SARS-CoV-2-encoded v-miRNAs target host genes that are involved in metabolic pathways, Table S1: List of primer sequences used for RT-qPCR, Table S2: List of antibodies, and dilutions used in this study, Table S3: List of top 10 v-miRNAs encoded by SARS-CoV-2 from Calu-3 and Vero E6 cells, Table S4: List of differentially expressed metabolic genes and their respective log2 fold changes.

## Abbreviations

ACO1	Aconitase 1
ATCC	American type culture collection
BCAS1	Brain enriched myelin associated protein 1
BNIP3L	BCL2 interacting protein 3 like
CO_2_	Carbon dioxide
CLDN10	Claudin 10
COVID-19	Corona virus disease -19
CXCL10	C-X-C motif chemokine ligand 10
DEG	Differentially expressed gene
DMBX1	Diencephalon/mesencephalon homeobox 1
DMEM	Dulbecco’s modified Eagle’s medium
DNA	Deoxyribonucleic acid
FBS	Fetal bovine serum
FDR	False discovery rate
GO	Gene ontology
HK	Hong Kong
IL-1β	Interleukin-1 beta
IL-6	Interleukin 6
KIF12	Kinesin family member 12
LPS	Lipopolysaccharide
MCP-1	Monocyte chemoattractant protein-1
MME	Membrane metalloendopeptidase
MOI	Multiplicity of infection
NEB	New England biolabs
NF-κB	Nuclear factor kappa B
NGS	Next-generation sequencing
NLRP3	Nucleotide oligomerization domain-like receptors family pyrin domain containing 3
ORF	Open reading frame
PBMC	Peripheral blood mononuclear cell
PBX1	Pre-B-cell leukemia transcription factor 1
PCR	Polymerase chain reaction
RNA	Ribonucleic acids
ROS 1	ROS proto-oncogene 1, receptor tyrosine kinase
RT	Reverse transcription
SARS-CoV-2	Severe acute respiratory syndrome coronavirus 2
SD	Standard deviation
SNCA	Synuclein alpha
SvRNA-N	Small viral RNA nucleocapsid
SYT12	Synaptotagmin 12
TFRC	Transferrin receptor protein 1
USA	United States of America
UPK1B	Uroplakin 1B
UTR	Untranslated region
V-miRNA	Viral microRNAs
