# Supplementary material for: Viral MicroRNAs Encoded by Nucleocapsid Gene of SARS-CoV-2 Are Detected during Infection, and Targeting Metabolic Pathways in Host Cells
Source: Cells. 2021 Jul 12;10(7):1762. doi: 10.3390/cells10071762 (PMC8307234; doi:10.3390/cells10071762)
Supplement: Supplementary file 1 [file cells-10-01762-s001.zip › cells-1268410-supplementary.pdf]

Supplementary Material

Table S1: List of primer sequences used for RT-qPCR

Gene	NCBI Ref Seq ID	Primer Sequences (5′ -> 3′)		Primer size (bp[1])	Primer location	Product size (bp)
ACO1	NM_001278352.2	FP[2]	AGAAGGGCAGACAGTTTACA	20	587 - 606	123
		RP[3]	TGAGCCAGGGGGAATAATC	19	709 - 691	
BCAS1	NM_003657.4	FP	TGCACAAGGAAGCAAAGTGC	20	20 - 39	137
		RP	AGCCCCTGTGGGAGTATCTT	20	156 - 137	
BCAT1	NM_001178094.2	FP	GCAAGATGGGAGGGAATTACG	21	916 - 936	178
		RP	GGAGGAGTTGCCAGTTCTTC	20	1093 - 1074	
BNIP3L	NM_001330491.2	FP	TGGCTTTGGGGCTAGGCAT	19	601 - 619	118
		RP	GACAATACACCACTTCACAGG	21	718 - 698	
CLDN10	NM_182848.4	FP	CAACAAAACACCCAGATACA	20	631 - 650	167
		RP	ATAACAAACTCAAGAGGCAG	20	797 - 778	
DMBX1	NM_001387775.1	FP	CGCCTGGCTGACATCATCTT	20	199 - 218	129
		RP	TGGGTAGTGAGTCTTCTGGA	20	327 - 308	
MME	NM_007287.4	FP	GCAACAGAAAACTGGGAGCAAAA	23	650 - 672	166
		RP	CAAGTCGAGGTTGGTCAATATGAA	24	815 - 792	
MMP11	NM_005940.5	FP	GATCGACTTCGCCAGGTACT	20	490 - 509	102
		RP	TCCCCTTCTCGGTGAGTCTT	20	591 - 572	
ROS1	NM_001378902.1	FP	CCTACCAACTGCTCCCTTTG	20	613 - 632	154
		RP	CGGTCTGGACACAGTCTTAG	20	766 - 747	
SNCA	NM_001375287.1	FP	GTGGCAACAGTGGCTGAGAA	20	667 - 686	184
		RP	GAATTCCTTCCTGTGGGGCT	20	850 - 831	
TFRC	NM_001313965.2	FP	TCATTCTTTGGACATGCTCATCTG	24	755 - 778	174
		RP	ACAGTCTCCTTCCATATTCCCAA	23	928 - 906	

[1]Base pair; [2]Forward primer; [3]Reverse primer

Table S2: List of antibodies, and dilutions used in this study

Antibody	Dilutions for WB[1]	Company	Catalogue Number
**NLRP3[2] (D2P5E), Rabbit mAb[3]**	1:1000	Cell Signaling Technology	13158S
**IL-1β[4] (D3U3E), Rabbit mAb**	1:1000	Cell Signaling Technology	12703
**β-actin (C4), Mouse monoclonal IgG1[5]**	1:1000	Santa Cruz Biotechnology	SC47778
**Goat anti-rabbit IgG, HRP[6]-linked Ab**	1:2000	Cell Signaling Technology	7074S
**Horse anti-mouse IgG, HRP-linked Ab**	1:2000	Cell Signaling Technology	7076S

[1]Western blotting; [2]Nucleotide oligomerization domain-like receptors family pyrin domain containing 3; [3]Monoclonal antibody; [4]Interleukin 1 beta; [5]Immunoglobulin G1; [6]Horse radish peroxidase

Table S3: List of top 10 v-miRNAs encoded by SARS-CoV-2 from Calu-3 and Vero E6 cells

V-miRNA[1]	Genome position	Size (bp[2])	Sequence 5′->3′	Coding gene	Mean Number of reads (%)
**95[3]_Calu-3**					
v-miRNA-5′UTR-44	44-63	20	CGATCTCTTGTAGATCTGTT	*5′UTR*	2.42
v-miRNA-ORF1-2836	2836-2857	22	CTCTGCCTATACAGTTGAACTC	*ORF1ab*	2.64
v-miRNA-ORF1-13613	13613-13631	19	AAAAGGACGAAGATGACAA	*ORF1ab*	1.52
v-miRNA-S-22423	22423-22443	21	TGCTGTAGACTGTGCACTTGA	*S*	1.39
v-miRNA-M-26823	26823-26843	21	AGACTGTTTGCGCGTACGCGT	*M*	1.56
v-miRNA-ORF7-27409	27409-27428	20	TTCTTGGCACTGATAACACT	*ORF7a*	2.14
v-miRNA-N-28612	28612-28631	20	AGGAACTGGGCCAGAAGCTG	*N*	1.49
v-miRNA-N-29094	29094-29112	19	TCGGCAGACGTGGTCCAGA	*N*	3.80
v-miRNA-N-29443	29443-29463	21	AACTGTGACTCTTCTTCCTGC	*N*	1.37
v-miRNA-ORF10-29597	29597-29616	20	TATAGTCTACTCTTGTGCAG	*ORF10*	1.84
**405[4]_Calu-3**					
v-miRNA-5′UTR-44	44-63	20	CGATCTCTTGTAGATCTGTT	*5′UTR*	2.57
v-miRNA-ORF1-4581	4581-4599	19	ACGATCTAAATGAAACTCT	*ORF1ab*	1.36
v-miRNA-ORF1-7458	7458-7479	22	TGCATGTTGTAGACGGTTGTAA	*ORF1ab*	1.01
v-miRNA-S-22423	22423-22443	21	TGCTGTAGACTGTGCACTTGA	*S*	3.25
v-miRNA-M-26823	26823-26843	21	AGACTGTTTGCGCGTACGCGT	*M*	2.36
v-miRNA-ORF7-27409	27409-27428	20	TTCTTGGCACTGATAACACT	*ORF7a*	1.50
v-miRNA-N-28612	28612-28631	20	AGGAACTGGGCCAGAAGCTG	*N*	3.14
v-miRNA-N-29094	29094-29112	19	TCGGCAGACGTGGTCCAGA	*N*	8.01
v-miRNA-N-29443	29443-29463	21	AACTGTGACTCTTCTTCCTGC	*N*	1.82
v-miRNA-ORF10-29597	29597-29616	20	TATAGTCTACTCTTGTGCAG	*ORF10*	1.96
**95_Vero E6**					
v-miRNA-ORF1-3109	3109-3127	19	GTATGGTACTGAAGATGAT	*ORF1ab*	1.34
v-miRNA-ORF1-3237	3237-3255	19	AAGACGGCAGTGAGGACAA	*ORF1ab*	0.91
v-miRNA-ORF1-7459	7459-7479	21	GCATGTTGTAGACGGTTGTAA	*ORF1ab*	1.07
v-miRNA-S-22423	22423-22443	21	TGCTGTAGACTGTGCACTTGA	*S*	2.34
v-miRNA-M-26823	26823-26843	21	AGACTGTTTGCGCGTACGCGT	*M*	1.50
v-miRNA-M-26990	26990-27011	22	AGGACGCTGTGACATCAAGGAC	*M*	1.17
v-miRNA-N-28386	28386-28405	20	AACAACGTCGGCCCCAAGGT	*N*	1.07
v-miRNA-N-28612	28612-28631	20	AGGAACTGGGCCAGAAGCTG	*N*	0.74
v-miRNA-N-29094	29094-29112	16	TCGGCAGACGTGGTCCAGA	*N*	1.48
v-miRNA-ORF10-29597	29597-29616	20	TATAGTCTACTCTTGTGCAG	*ORF10*	1.15
**405_Vero E6**					
v-miRNA-ORF1-3109	3109-3127	19	GTATGGTACTGAAGATGAT	*ORF1ab*	1.65
v-miRNA-ORF1-3237	3237-3255	19	AAGACGGCAGTGAGGACAA	*ORF1ab*	1.74
v-miRNA-ORF1-7459	7459-7479	21	GCATGTTGTAGACGGTTGTAA	*ORF1ab*	1.37
v-miRNA-S-22423	22423-22443	21	TGCTGTAGACTGTGCACTTGA	*S*	13.5
v-miRNA-ORF3-25528	25528-25547	20	CTTATTGTTGGCGTTGCACT	*ORF3a*	1.60
v-miRNA-M-26820	26820-26841	22	TTCAGACTGTTTGCGCGTACGC	*M*	2.74
v-miRNA-M-26990	26990-27011	22	AGGACGCTGTGACATCAAGGAC	*M*	2.55
v-miRNA-N-28612	28612-28631	20	AGGAACTGGGCCAGAAGCTG	*N*	0.34
v-miRNA-N-29136	29136-29156	21	ACCAGGAACTAATCAGACAAG	*N*	1.03
v-miRNA-3′UTR-29688	29688-29708	21	GTGTGTAACATTAGGGAGGAC	*3′UTR*	2.16

[1]Viral microRNA; [2]Base pair; [3]SARS-CoV-2, clone Hong Kong-95; [4]SARS-CoV-2, clone Hong Kong-405

Table S4: List of differentially expressed metabolic genes and their respective log_2 fold changes

Target gene	Log_2FC[1]	*P* value	Key functions in the metabolic pathways
ACO1	-1.6	$3.93E^{-20}$	Involved in TCA cycle and interacts with mRNA to control the levels of iron inside cells, and carbon metabolism
BCAS1	-2	$2.47E^{-3}$	Plays a role in myelinating oligodendrocytes related to multiple sclerosis lesions
BCAT1	-1.9	$8.94E^{-9}$	Controls metabolic reprogramming, and promotes cell proliferation through amino acid catabolism
BNIP3L	-1.4	$5.05E^{-21}$	Apoptosis modulation; signaling and gene expression
CLDN10	-1.3	$2.87E^{-7}$	Regulates blood-brain barrier, and immune cell transmigration
DMBX1	-2.3	$4.33E^{-5}$	Plays a role in brain and sensory organ development, and in normal regulation of energy homeostasis and behavior
KIF12	-1.2	$1.44E^{-6}$	Antioxidant activity; intracellular transport; cell division
MME	-1.5	$1.56E^{-7}$	A neutral endopeptidase that cleaves and inactivates peptides, including hormones
MMP11	-2.4	$2.14E^{-19}$	Plays a role in controlling energy metabolism; extracellular matrix (ECM) remodeling/degradations; metabolic reprogramming
PBX1	-1.2	$3.43E^{-3}$	Promotes cell proliferation and plays oncogenic activity; regulate osteogenesis; enhances cellular metabolism
ROS1	-2.2	$6.38E^{-5}$	Involved in cell differentiation and has oncogenic role
SNCA	-2.8	$8.04E^{-15}$	Enhances glucose uptake and utilization; integrate presynaptic signaling, and membrane trafficking
SYT12	-1.3	$2.57E^{-3}$	Mediate calcium-dependent regulation of membrane trafficking in synaptic transmission
TFRC	-1.1	$1.22E^{-151}$	Required for erythropoiesis and neurologic development; iron transport
UPK1B	-1.2	$2.52E^{-16}$	Mediate signal transduction events that play a role in the regulation of cell development, activation, growth and motility

[1]Log_2 Fold Change

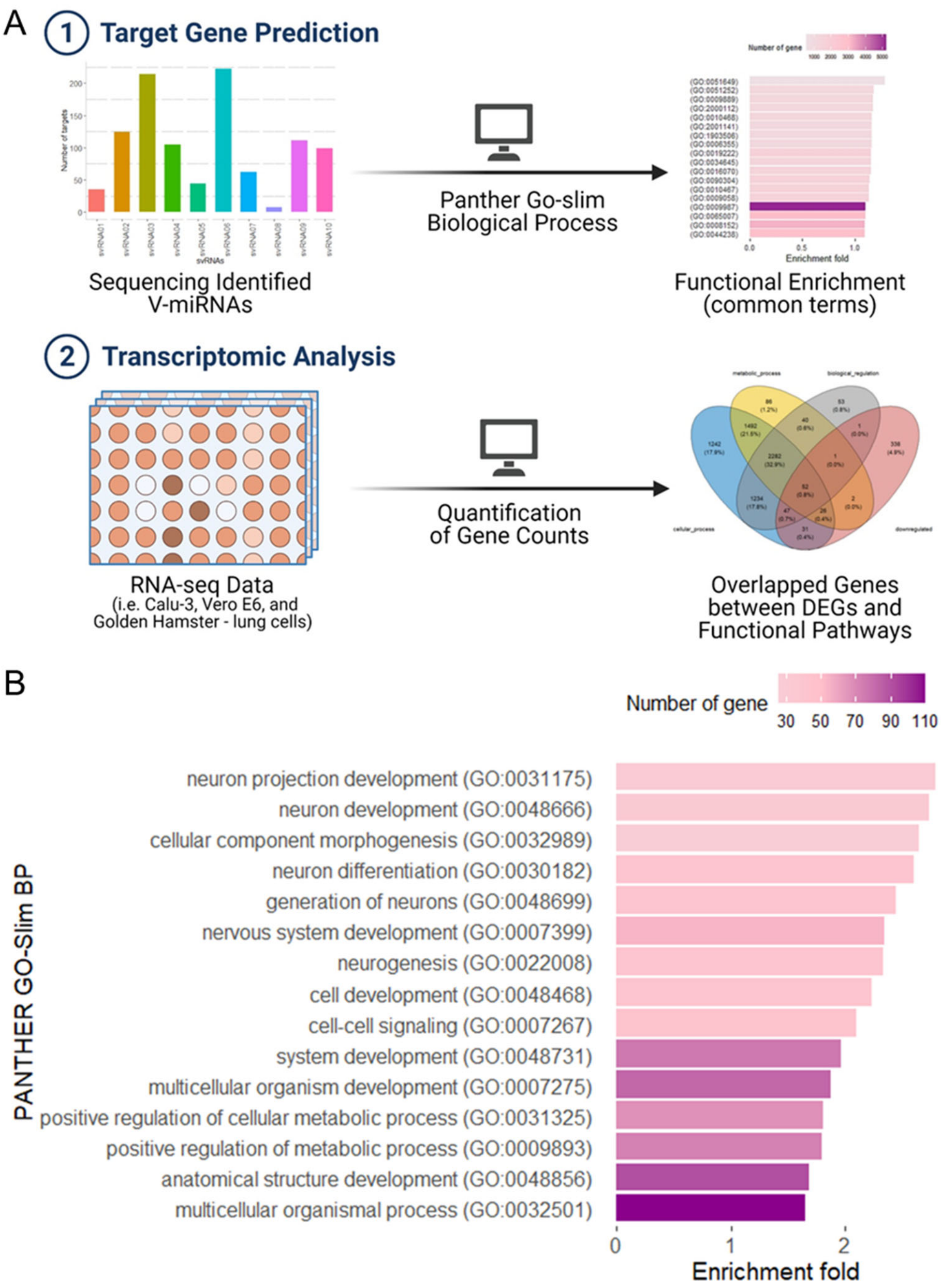

Figure S1. SARS-CoV-2-encoded v-miRNAs target host genes that are involved in metabolic pathways. (**A**) GO analysis indicates the main enrichment terms targeted by SARS-CoV-2-encoded v-miRNAs. (1) Shows the target gene prediction and selected common terms; (2) Shows transcriptomic analysis, and genes overlapping between DEGs and functional pathways. (**B**) Enriched common GO pathways for v-miRNA potential targets predicted by using the overlap of miRDB and Diana. The most significant 15 GO terms are selected for the presentation. The color represents the number of predicted genes in each GO term.
